# A bibliometric analysis of classic publications in web of science category of orthopedics

**DOI:** 10.1186/s13018-019-1247-1

**Published:** 2019-07-19

**Authors:** Yunzhu Li, Gang Xu, Xiao Long, Yuh-Shan Ho

**Affiliations:** 1 Department of Plastic Surgery, Peking Union Medical College (PUMC) Hospital, PUMC and Chinese Academy of Medical Sciences, Beijing, China; 2 Department of Liver Surgery, Peking Union Medical College (PUMC) Hospital, PUMC and Chinese Academy of Medical Sciences, Beijing, China; 3 Trend Research Centre, Asia University, No. 500, Lioufeng Road, Wufeng, Taichung Country, 41354 Taiwan; 4 Peking Union Medical College Hospital, No. 1, Shuaifuyuan, Dongcheng District, Beijing, China

**Keywords:** Orthopedics, Bibliometric, Classic publications, Web of science

## Abstract

**Background:**

The past century has witnessed the rapid development of operation technique, surgical instruments, and knowledge of the diseases in orthopedics. In the academic history, a number of classic papers boosted the advancement for surgery. In this paper, we performed a bibliometric analysis, aiming to determine the most influential studies within the field.

**Methods:**

Articles were searched from the publication year of 1900 to 2016 according to the Science Citation Index Expanded database of the Clarivate Analytics Web of Science Core Collection database. Two citation indicators *TC*_year_ and *C*_year_ were employed to characterize the classic articles and the articles were identified and analyzed.

**Results:**

A total of 30 classic articles with *TC*_2016_ ≥ 1000 in Web of Science category of orthopedics were identified, all written in English between 1961 and 2007 by nine countries. The minimal value of *TC*_2016_ was 1010; the maximum 3570; and the average 1591. Thirty classic articles were published in eight journals that were listed in the Web of Science category of orthopedics in 2016, and in two other orthopedics journals that were no longer tracked by Web of Science category of orthopedics as of 2016. Among the top 10 cited articles in both *TC*_2016_ and *C*_2016_, five articles barely received attention in the first few years after their publication, while they became cited more and more frequently in the last decade.

**Conclusion:**

This study evaluated the development and trend of orthopedics research by adopting bibliometric analysis. It serves as a guide for investigators in the future research.

## Background

The modern term orthopedics derives from the older word orthopaedia, title of a book published in 1741 by Nicholas Andry [1]. Two Greek words orthos and paedios serve as roots for orthopedic surgery. The former one means straight and free of deformity and the latter one means a child [2]. Orthopedic surgery demonstrates a rapid progress with several recent advances noted within orthopedic subspecialties [3–5], basic science [6], and clinical research [7]. Bibliometrics is a widely used tool to map the literature around a research field. It can help us to gain insight into the research focuses and future development of trends in orthopedic surgery. The citation number of a published article approximately reflects the popularity of the study and indicates the significance of the article in a certain field [8]. A thorough bibliometric analysis of classic articles helps investigators efficiently learns the history of developments and future directions of a research field. In this study, classic articles were identified and their characteristics were analyzed based on the bibliometric analysis method in the hope that it may guide investigators in this field.

## Materials and methods

Our study was based on the Science Citation Index Expanded (SCI-EXPANDED) database of the Clarivate Analytics (formerly known as the Thomson Reuters and the Institute for Scientific Information) Web of Science (WOS) Core Collection database. According to Journal Citation Reports (JCR) of 2016 (InCites Journal Citation Reports dataset updated September 09, 2017), it indexes 8879 journals with citation references across 177 WOS categories in SCI-EXPANDED. In total, 302,299 documents (including 227,023 articles) were found in WOS category of orthopedics from the publication of 1900 to 2016 based on SCI-EXPANDED (updated on March 12, 2018). Two citation indicators *TC*_year_ and *C*_year_ were employed to characterize the classic articles. *TC*_year_ is the total citation number from WOS Core Collection since publication to the end of the most recent year [9, 10]. *C*_year_ is the number of citations in the most recent year. *C*_2016_ means the number of citation in 2016. *TC*_year_ ≥ 1000 was used to retrieve the classic articles [11–13]. We inserted all the data for each article for each year into spreadsheet software, and manipulated them using Microsoft Excel2016 [14, 15]. In addition, all hard copies of the 32 classic publications were found to check analysis information. Affiliations in England, Scotland, Northern Ireland, and Wales were reclassified as being from the United Kingdom (UK) [16].

## Results and discussion

### Document type and language of publication

Analysis of document types and their citations per publication was earlier proposed [17]. A total of 32 classic publications (0.011% of 302,299 documents) with *TC*_2016_ ≥ 1000 in WOS of orthopedics were found within two document types indexed in the WOS. Thirty classic publications were found to be document type of article including three of them belonging to both document types of article and proceedings paper. Two were published as document type of review. A review entitled “OARSI recommendations for the management of hip and knee osteoarthritis, Part II: OARSI evidence-based, expert consensus guidelines” [18] was the only classic document published in the latest year of 2008 in orthopedics field with *TC*_2016_ of 1394. Only articles were used for subsequent analysis because they included complete research ideas and results [19]. As a result, we identified 30 classic articles (0.013% of 227,023 articles) in the category of orthopedics, all of which were written in English. Such low percentage of classic publications can be also found, for example 0.048% and 0.063% of all documents in WOS categories of neurosciences [20] and psychology [12] respectively as well as 0.046% and 0.0049% of all articles in WOS categories of neurosciences [20] and surgery [11] respectively.

### Publication years

**Fig. 1:**
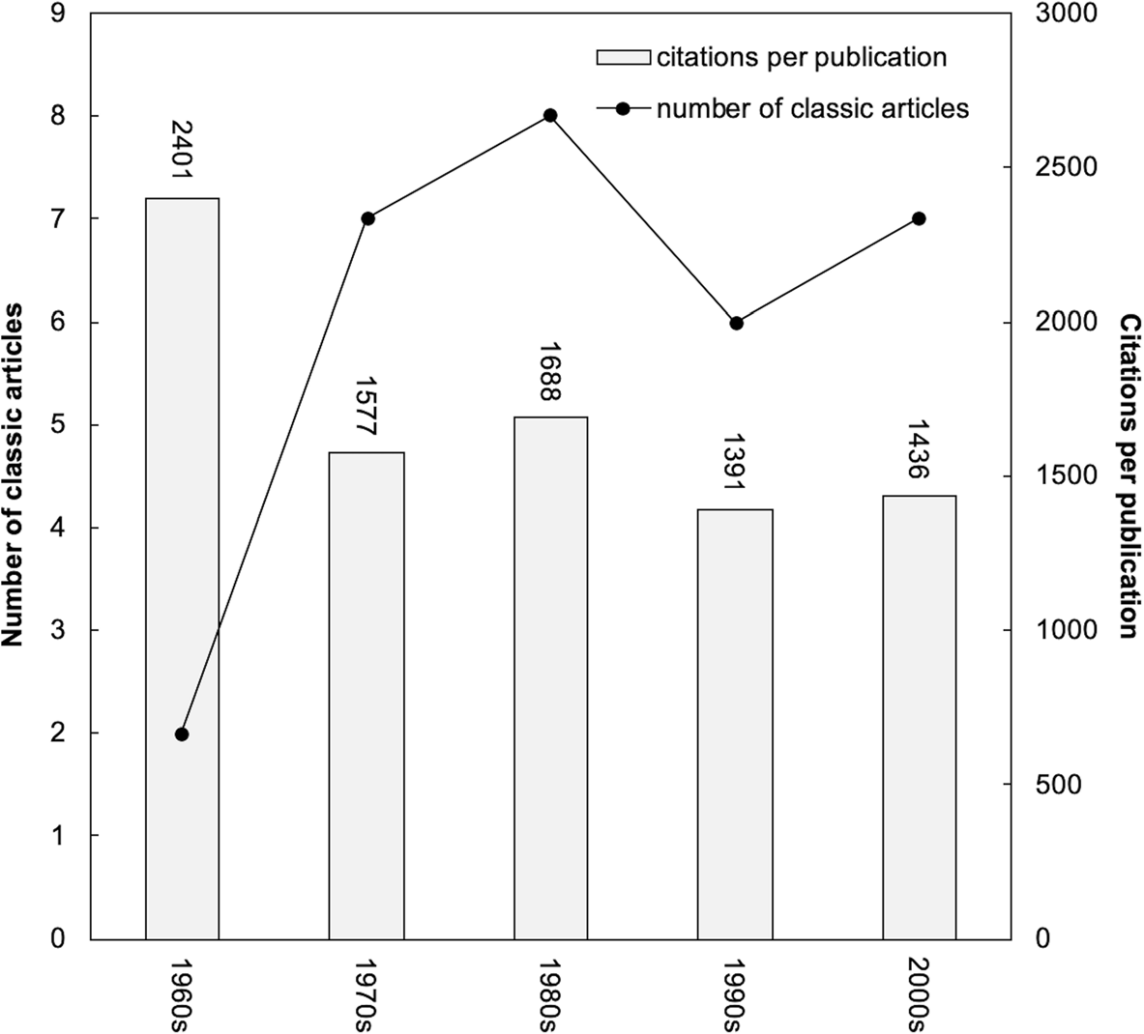
Number of classic articles and citations per publication by decade

In recent years, Ho’s group proposed a relationship between total number of classic articles in a year (*TP*) and their citations per publication (*CPP*_2016_ = *TC*_2016_/*TP*) by the decades in a WOS category as a unique indicator, for example WOS category of surgery [11], psychology [12], and neurosciences [20]. Thirty classic articles in WOS category of orthopedics were published between 1961 and 2007. The maximum value of *TC*_2016_ was 3570, the minimum 1010, and the average 1591. Figure 1 shows the distribution of these 30 classic articles over the decades, and their citations per publication (*CPP*_2016_). The 30 classic articles received a total of 47,735 citations. Only two classic articles were found in the decade of the 1960s, and no classic article was identified in the most recent decades. The 1980s was the most prolific period in terms of classic articles in orthopedics, which was different from WOS categories of the 1970s in surgery [11], the 1970s in psychology [12], and the 1990s in neurosciences [20]. Besides, the decade of the 1960s had the highest *CPP*_2016_ of 2401. The earliest classic article in orthopedics field was “The etiology of chondromalacia patellae” [21] published in the *Journal of Bone and Joint Surgery*-*British Volume* by Outerbridge from Royal Columbian Hospital in Canada in 1961 with *TC*_2016_ of 1331 (ranked 19th) and *C*_2016_ of 78 (ranked 22th). The latest classic article was found in 2007 by five authors from Exponent Inc., entitled “Projections of primary and revision hip and knee arthroplasty in the United States from 2005 to 2030” [22] in the *Journal of Bone and Joint Surgery*-*American Volume* with *TC*_2016_ of 2012 (ranked 6th) and *C*_2016_ of 411 (ranked 1st).

### Journals

A total of 76 journals were listed in the WOS category of orthopedics in 2016. The 30 classic articles were published in eight of these journals (11% of 76 journals), and in two other orthopedics journals that were no longer tracked by Web of Science category of orthopedics as of 2016 (Table 1). The *Journal of Bone and Joint Surgery*-*American Volume* with *IF*_2016_ of 4.840 (rank 2nd of 76 orthopedics journals) published the largest number of classic articles with nine articles (30% of 30 classic articles), followed by *Clinical Orthopedics and Related Research* with seven. *American Journal of Sports Medicine* had the highest *IF*_2016_ with only one classic article. *Connective Tissue Research* with *IF*_2016_ of 1.832 (ranked 33th) also had only one classic article. The *Journal of Bone and Joint Surgery*-*British Volume* (*IF*_2014_ = 3.309) and *Acta Orthopaedica Scandinavica* (*IF*_2004_ = 1.108) were not in SCI-EXPANDED in 2014 and 2004 respectively.

**Table 1:** The ten journals with classic articles in Web of Science category of orthopedics

Journal	*TP* (%)	*IF*_2016_ (rank*)
Journal of Bone and Joint Surgery-American Volume	9 (30)	4.840 (2)
Clinical Orthopedics and Related Research	7 (23)	3.897 (6)
Spine	4 (13)	2.499 (20)
Physical Therapy	3 (10)	2.764 (14)
Journal of Orthopedic Research	2 (6.7)	2.692 (16)
Acta Orthopaedica Scandinavica	1 (3.3)	1.108 in 2004
American Journal of Sports Medicine	1 (3.3)	5.673 (1)
Connective Tissue Research	1 (3.3)	1.832 (33)
Foot & Ankle International	1 (3.3)	1.872 (32)
Journal of Bone and Joint Surgery-British Volume	1 (3.3)	3.309 in 2014

*TP* total number of classic articles, *IF*_2016_ impact factor for 2016; *: rank of *IF*_2016_ in Web of Science category of orthopedics

### Countries, institutions, and authors

There were 30 classic articles in WOS category of orthopedics by nine countries. Twenty-seven articles (90% of 30 articles) were completed in a single country from five countries and three (10%) were completed international-collaboratively from six countries. The USA took the first place by total classic articles with 18 (60% of 30 articles), followed by the UK (six articles; 20% of 30 articles), Sweden (three; 10%), Canada (two; 6.7%), and one for each of Australia, Brazil, France, Japan, and Switzerland respectively. The USA also published 16 of 27 single-country articles, two of three internationally collaborative articles, 16 of 30 first author articles, 14 of 28 corresponding articles, and four of five single-author articles.

In total, 18 (60% of 30 articles) articles were completed in a single institution from 17 institutions and 12 (40%) were completed inter-institutional-collaboratively from 31 institutions. Only two institutions such as Case Western Reserve University in USA and Linköping University Hospital in Sweden published two classic articles in WOS category of orthopedics. Other 45 institutions had only one classic article. Linköping University Hospital was also the only one that published two single institution classic articles, first author articles, and corresponding author articles. Twenty-two of the 47 classic institutions were located in the USA followed by nine from the UK, five from Canada, three from Sweden, three from Australia, two from Japan, and one from Switzerland, France, and Brazil respectively.

Among the 91 classic authors of the 20 classic articles in WOS category of orthopedics, only A.I. Caplan from Case Western Reserve University in the USA and J. Lysholm from Linköping University Hospital in Sweden published two classic articles including one first author and one corresponding author articles. A.I. Caplan also published one single author classic article. Other 89 authors published only one classic article (Table 2).

**Table 2:** Authors with classic articles in Web of Science category of orthopedics

Author	Institution	Rank (TP)	Rank (FP)	Rank (RP)	Rank (SP)
A.I. Caplan	Case Western Reserve University, USA	1 (2)	1 (1)	1 (1)	1 (1)
J. Lysholm	Linkoping University Hospital, Sweden	1 (2)	1 (1)	1 (1)	N/A
R.S. Adelaar	N/A	3 (1)	N/A	N/A	N/A
T. Albrektsson	University of Gothenburg, Sweden	3 (1)	1 (1)	1 (1)	N/A
I.J. Alexander	N/A	3 (1)	N/A	N/A	N/A
H.C. Amstutz	N/A	3 (1)	N/A	N/A	N/A
J.T. Anderson	N/A	3 (1)	N/A	N/A	N/A
A.J. Barrett	N/A	3 (1)	N/A	N/A	N/A
D.E. Beaton	Institute for Work and Health, Canada	3 (1)	1 (1)	1 (1)	N/A
M. Beck	N/A	3 (1)	N/A	N/A	N/A
S.D. Boden	George Washington University, USA	3 (1)	1 (1)	1 (1)	N/A
R.W. Bohannon	Cape Fear Valley Medical Center, USA	3 (1)	1 (1)	1 (1)	N/A
C. Bombardier	N/A	3 (1)	N/A	N/A	N/A
J.W. Bowerman	N/A	3 (1)	N/A	N/A	N/A
P.I. Branemark	N/A	3 (1)	N/A	N/A	N/A
A.F. Brooker	Johns Hopkins Hospital, USA	3 (1)	1 (1)	N/A	N/A
D.R. Carter	University of Washington, USA	3 (1)	1 (1)	N/A	N/A
J. Charnley	Charnley, UK	3 (1)	N/A	1 (1)	N/A
C.R. Constant	Addenbrooke’s Hospital, UK	3 (1)	1 (1)	1 (1)	N/A
D.O. Davis	N/A	3 (1)	N/A	N/A	N/A
J.G. Delee	Wrightington Hospital, UK	3 (1)	1 (1)	N/A	N/A
T.S. Dina	N/A	3 (1)	N/A	N/A	N/A
H. Dorfman	N/A	3 (1)	N/A	N/A	N/A
L.D. Dorr	N/A	3 (1)	N/A	N/A	N/A
W. Dunham	N/A	3 (1)	N/A	N/A	N/A
M. Elkins	N/A	3 (1)	N/A	N/A	N/A
W.F. Enneking	University of Florida, USA	3 (1)	1 (1)	1 (1)	N/A
J.C.T. Fairbank	Nuffield Orthopedic Centre, UK	3 (1)	1 (1)	1 (1)	N/A
R.W. Farndale	Strangeways Research Laboratory, UK	3 (1)	1 (1)	1 (1)	N/A
M.B. Ferraz	N/A	3 (1)	N/A	N/A	N/A
R. Ganz	University of Berne, Switzerland	3 (1)	1 (1)	1 (1)	N/A
M.C. Gebhardt	N/A	3 (1)	N/A	N/A	N/A
J. Gillquist	N/A	3 (1)	N/A	N/A	N/A
V.M. Goldberg	N/A	3 (1)	N/A	N/A	N/A
T. Goto	N/A	3 (1)	N/A	N/A	N/A
T.A. Gruen	Univ Calif Los Angeles, USA	3 (1)	1 (1)	N/A	N/A
F. Guillemin	N/A	3 (1)	N/A	N/A	N/A
R.B. Gustilo	Hennepin County Medical Center, USA	3 (1)	1 (1)	N/A	N/A
M. Halpern	N/A	3 (1)	N/A	N/A	N/A
H.A. Hansson	N/A	3 (1)	N/A	N/A	N/A
W.H. Harris	Massachusetts General Hospital, USA	3 (1)	1 (1)	1 (1)	1 (1)
W.C. Hayes	N/A	3 (1)	N/A	N/A	N/A
R.D. Herbert	N/A	3 (1)	N/A	N/A	N/A
J.N. Insall	Hospital for Special Surgery, USA	3 (1)	1 (1)	1 (1)	N/A
M.P. Kadaba	Helen Hayes Hospital, USA	3 (1)	1 (1)	1 (1)	N/A
H.B. Kitaoka	Mayo Clinic & Mayo Foundation, USA	3 (1)	1 (1)	1 (1)	N/A
S. Kurtz	Exponent Inc., USA	3 (1)	1 (1)	1 (1)	N/A
E. Lau	N/A	3 (1)	N/A	N/A	N/A
M. Leunig	N/A	3 (1)	N/A	N/A	N/A
J. Lindstrom	N/A	3 (1)	N/A	N/A	N/A
L. Lippiell	N/A	3 (1)	N/A	N/A	N/A
C.G. Maher	University of Sydney, Australia	3 (1)	1 (1)	1 (1)	N/A
M. Malawar	N/A	3 (1)	N/A	N/A	N/A
H.J. Mankin	Hospital for Joint Diseases, USA	3 (1)	1 (1)	N/A	N/A
J.M. Mansour	N/A	3 (1)	N/A	N/A	N/A
G.M. Mcneice	N/A	3 (1)	N/A	N/A	N/A
R. Morris	N/A	3 (1)	N/A	N/A	N/A
A.M. Moseley	N/A	3 (1)	N/A	N/A	N/A
F. Mowat	N/A	3 (1)	N/A	N/A	N/A
A.H.G. Murley	N/A	3 (1)	N/A	N/A	N/A
M.S. Myerson	N/A	3 (1)	N/A	N/A	N/A
C.S. Neer	Columbia University, USA	3 (1)	1 (1)	1 (1)	1 (1)
H. Notzli	N/A	3 (1)	N/A	N/A	N/A
J.A. Nunley	N/A	3 (1)	N/A	N/A	N/A
K. Ong	N/A	3 (1)	N/A	N/A	N/A
R.E. Outerbridge	Royal Columbian Hospital, Canada	3 (1)	1 (1)	1 (1)	1 (1)
J. Parvizi	N/A	3 (1)	N/A	N/A	N/A
N.J. Patronas	N/A	3 (1)	N/A	N/A	N/A
S.J. Pineda	N/A	3 (1)	N/A	N/A	N/A
D.J. Pritchard	N/A	3 (1)	N/A	N/A	N/A
P.B. Pynsent	N/A	3 (1)	N/A	N/A	N/A
H.K. Ramakrishnan	N/A	3 (1)	N/A	N/A	N/A
L.H. Riley	N/A	3 (1)	N/A	N/A	N/A
R.A. Robinson	N/A	3 (1)	N/A	N/A	N/A
M. Roland	St. Thomas’ Hospital Medical School, UK	3 (1)	1 (1)	1 (1)	N/A
M. Sanders	N/A	3 (1)	N/A	N/A	N/A
C.A. Sayers	N/A	3 (1)	N/A	N/A	N/A
R.D. Scott	N/A	3 (1)	N/A	N/A	N/A
W.N. Scott	N/A	3 (1)	N/A	N/A	N/A
C. Sherrington	N/A	3 (1)	N/A	N/A	N/A
K.A. Siebenrock	N/A	3 (1)	N/A	N/A	N/A
J. Sim	Keele University, UK	3 (1)	1 (1)	1 (1)	N/A
M.B. Smith	N/A	3 (1)	N/A	N/A	N/A
Y. Tegner	Linkoping University Hospital, Sweden	3 (1)	1 (1)	1 (1)	N/A
S. Wakitani	Osaka University Hospital, Japan	3 (1)	1 (1)	1 (1)	N/A
J.E. Ware	Quality Metric Inc., USA	3 (1)	1 (1)	1 (1)	1 (1)
S.W. Wiesel	N/A	3 (1)	N/A	N/A	N/A
M.E. Wootten	N/A	3 (1)	N/A	N/A	N/A
C.C. Wright	N/A	3 (1)	N/A	N/A	N/A
R.G. Young	N/A	3 (1)	N/A	N/A	N/A

*TP* total number of classic articles, *FP* number of first author classic articles, *RP* number of corresponding author classic articles, *SP* number of single author classic articles

### Citation history of classic articles

Table 3 shows the 30 classic articles in WOS category of orthopedics with both citation numbers and rankings for *TC*_2016_ and *C*_2016_. Total citations indicated high impact or visibility of an article in a research field. Due to the citations of publications in WOS Core Collection were updated weekly, the total citation number an article has since its publication to the end of 2016 (*TC*_2016_) was utilized [9, 10]. The advantage of *TC*_2016_ is that they remain invariable and ensure repeatability compared with the index of citation from WOS Core Collection [12]. The history of a publication’s citations with time has long been studied [51]. The citation history shows characteristics of the influence of an article after its publication. The highly cited articles would not always have high influence or visibility in research society [52]. Five of the top 10 articles (*TC*_2016_ > 1800) still have a *C*_2013_ ranked in the top 10.

**Table 3:** The 30 classic articles in Web of Science category of orthopedics

Rank (*TC*_2016_)	Rank (*C*_2016_)	Article author	Article title
1 (3470)	3 (253)	Harris (1969) [23]	Traumatic arthritis of hip after dislocation and acetabular fractures: treatment by mold arthroplasty: an end-result study using a new method of result evaluation
2 (2169)	5 (197)	Bohannon and Smith (1987) [24]	Interrater reliability of a modified Ashworth scale of muscle spasticity
3 (2161)	9 (161)	Insall et al. (1989) [25]	Rationale of the knee society clinical rating system
4 (2115)	13 (150)	Caplan (1991) [26]	Mesenchymal stem cells
5 (2058)	16 (107)	Constant and Murley (1987) [27]	A clinical method of functional assessment of the shoulder
6 (2012)	1 (411)	Kurtz et al. (2007) [22]	Projections of primary and revision hip and knee arthroplasty in the United States from 2005 to 2030
7 (1870)	2 (363)	Beaton et al. (2000) [28]	Guidelines for the process of cross-cultural adaptation of self-report measures
8 (1817)	26 (53)	Brooker et al. (1973) [29]	Ectopic ossification following total hip replacement: Incidence and a method of classification
9 (1816)	18 (97)	Gustilo and Anderson (1976) [30]	Prevention of infection in the treatment of one thousand and twenty-five open fractures of long bones: Retrospective and prospective analyses
10 (1811)	17 (99)	Roland and Morris (1983) [31]	A study of the natural history of back pain. Part I. Development of a reliable and sensitive measure of disability in low-back pain
11 (1771)	15 (110)	Gruen et al. (1979) [32]	“Modes of failure” of cemented stem-type femoral components: A radiographic analysis of loosening
12 (1764)	8 (163)	Kitaoka et al. (1994) [33]	Clinical rating systems for the ankle-hindfoot, midfoot, hallux, and lesser toes
13 (1674)	10 (159)	Tegner and Lysholm (1985) [34]	Rating systems in the evaluation of knee ligament injuries
14 (1666)	24 (67)	Mankin et al. (1971) [35]	Biochemical and metabolic abnormalities in articular cartilage from osteo-arthritic human hips. II. Correlation of morphology with biochemical and metabolic data
15 (1440)	19 (85)	Delee and Charnley (1976) [36]	Radiological demarcation of cemented sockets in total hip replacement
16 (1394)	4 (229)	Fairbank and Pynsent (2000) [37]	The Oswestry Disability Index
17 (1367)	21 (80)	Albrektsson et al. (1981) [38]	Osseointegrated titanium implants: Requirements for ensuring a long-lasting, direct bone-to-implant anchorage in man
18 (1365)	7 (177)	Ware (2000) [39]	SF-36 health survey update
19 (1331)	22 (78)	Outerbridge (1961) [21]	The etiology of chondromalacia patellae
20 (1306)	27 (46)	Neer (1972) [40]	Anterior acromioplasty for chronic impingement syndrome in shoulder: A preliminary report
21 (1226)	12 (151)	Ganz et al. (2003) [41]	Femoroacetabular impingement: A cause for osteoarthritis of the hip
22 (1225)	23 (74)	Lysholm and Gillquist (1982) [42]	Evaluation of knee ligament surgery results with special emphasis on use of a scoring scale
23 (1220)	28 (39)	Carter and Hayes (1977) [43]	The compressive behavior of bone as a two-phase porous structure
24 (1201)	24 (67)	Boden et al. (1990) [44]	Abnormal magnetic-resonance scans of the lumbar spine in asymptomatic subjects: A prospective investigation
25 (1179)	14 (112)	Kadaba et al. (1990) [45]	Measurement of lower extremity kinematics during level walking
26 (1176)	6 (190)	Sim and Wright (2005) [46]	The kappa statistic in reliability studies: Use, interpretation, and sample size requirements
27 (1059)	20 (82)	Enneking et al. (1993) [47]	A system for the functional evaluation of reconstructive procedures after surgical treatment of tumors of the musculoskeletal system
28 (1037)	30 (32)	Farndale et al. (1982) [48]	A direct spectrophotometric micro-assay for sulfated glycosaminoglycans in cartilage cultures
29 (1026)	28 (39)	Wakitani et al. (1994) [49]	Mesenchymal cell-based repair of large, full-thickness defects of articular cartilage
30 (1010)	11 (153)	Maher et al. (2003) [50]	Reliability of the PEDro scale for rating quality of randomized controlled trials

*TC*_2016_ total citations from Web of Science Core Collection since publication to the end of 2016, *C*_2016_ citations in 2016 only

**Fig. 2:**
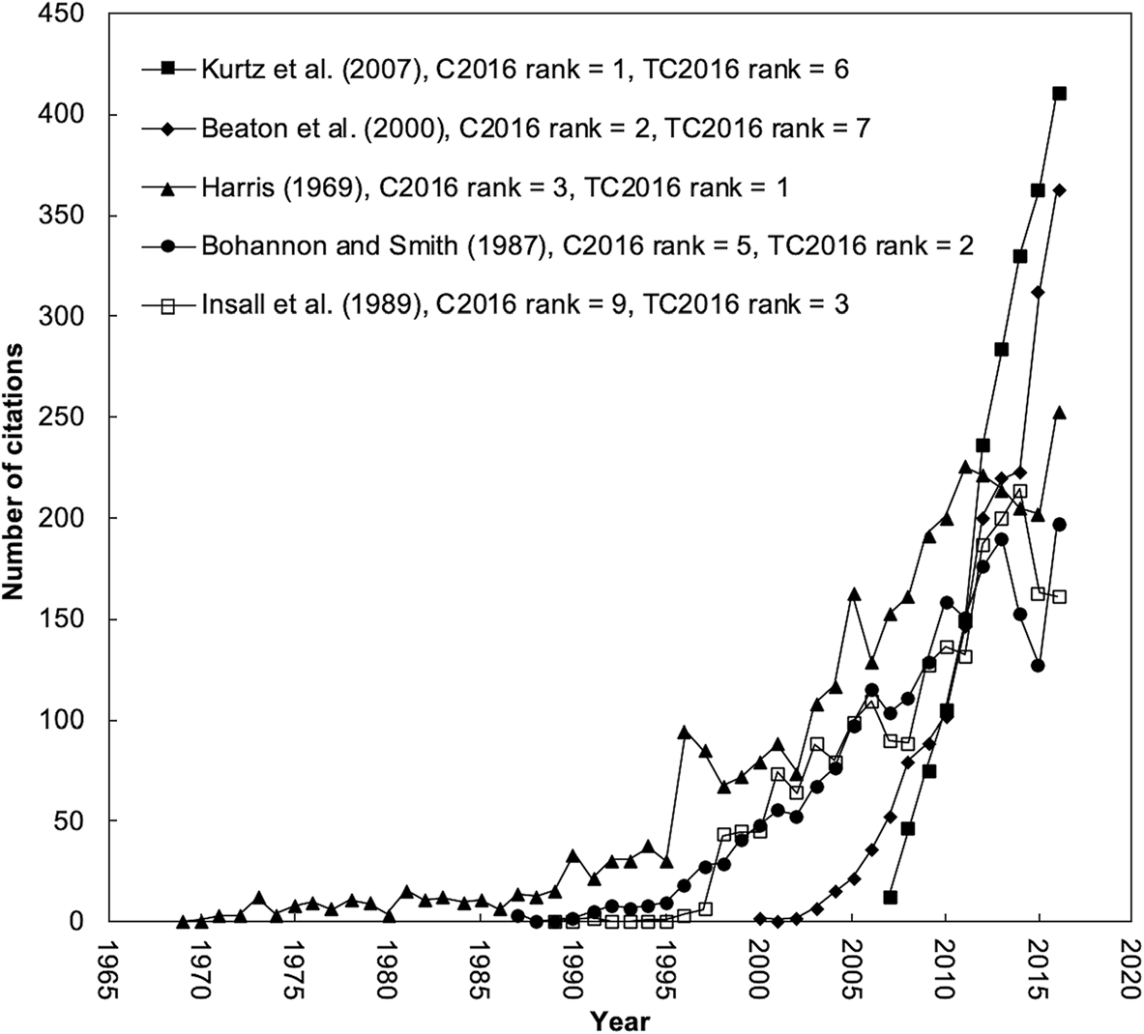
Citation history of the five classic articles ranked in the top 10 of both *TC*_2016_ and *C*_2016_

Figure 2 shows the citation history of classic articles that were ranked among the top 10 in both *TC*_2016_ and *C*_2016_. Although some recently published articles within the past few years had great potential, they did not have a high *TC*_2016_. Thus indicator of *C*_2016_ would be interesting to show high impact in 2016. A typical example is the article entitled “Projections of primary and revision hip and knee arthroplasty in the United States from 2005 to 2030” [22] which was the most impact classic article in 2016 with *C*_2016_ of 411. A sharply increasing trend of citations can be found in this article. Similarly, the article entitled “Guidelines for the process of cross-cultural adaptation of self-report measures” had the same impact trend as the article by Beaton et al. [28] in the last decade. Other three articles including Harris et al. [23], Bohannon and Smith [24], and Insall et al. [25] had low citations after their publication and then had an increasing trend in the last 10 years. Classic articles by Fairbank and Pynsent [37], Ware [39], Ganz et al. [41], Sim and Wright [46], and Maher et al. [50] also had sharply increasing citations after publication. Table 4 reveals characteristic of highly cited and the most impact classic articles. The five classic articles were highlighted as follows:

- Projections of primary and revision hip and knee arthroplasty in the USA from 2005 to 2030 [22] with *C*_2016_ of 411 and *TC*_2016_ of 2012.

**Table 4:** The characteristic of highly cited and the most impact classic articles

Rank (TC2016)	Rank (C2016)	References (year)	Country	Affiliation	Article title
6 (2012)	1 (411)	Kurtz et al. (2007) [22]	USA	Exponent Inc.	Projections of primary and revision hip and knee arthroplasty in the United States from 2005 to 2030
7 (1870)	2 (363)	Beaton et al. (2000) [28]	Canada	Michael’s Hospital	Guidelines for the process of cross-cultural adaptation of self-report measures
1 (3470)	3 (253)	Harris (1969) [23]	USA	Massachusetts General Hospital	Traumatic arthritis of hip after dislocation and acetabular fractures: treatment by mold arthroplasty: an end-result study using a new method of result evaluation
2 (2169)	5 (197)	Bohannon and Smith.(1987) [24]	USA	Southeastern Regional Rehabilitation Center	Interrater reliability of a modified Ashworth scale of muscle spasticity
3 (2161)	9 (161)	Insall et al.(1989) [25]	USA	Hospital for Special Surgery	Rationale of the knee society clinical rating system

*TC*_2016_ total citations from Web of Science Core Collection since publication to the end of 2016, *C*_2016_ citations in 2016 only

Based on NIS, the study collected a substantially large number of discharge records, and revealed the information of the demand for primary and revision hip and knee arthroplasties in the USA through 2030 for the first time. It helped to quantify the expected number of hip and knee revision arthroplasties in the future. It also laid the necessary foundation for subsequent cost-benefit analysis nationally, to measure the increasing societal impact of revision arthroplasty in the USA.

- Guidelines for the process of cross-cultural adaptation of self-report measures [28] with *C*_2016_ of 363 and *TC*_2016_ of 1870.

With the increasing number of multinational and multicultural research projects, there is a growing need to adapt the language of health status measures. The term “cross-cultural adaptation” is used to describe a process that involves both language (translation) and cultural adaptation issues in the process of preparing a questionnaire. This paper firstly presented a guideline for the process of cross-cultural adaptation of self-report measures, allowing equal efforts to collect data in cross-national studies and to avoid the selection bias.

- Traumatic arthritis of hip after dislocation and acetabular fractures: treatment by mold arthroplasty: an end-result study using a new method of result evaluation [23] with *C*_2016_ of 253 and *TC*_2016_ of 3470.

The Harris Hip Score was initially introduced in this paper as a research tool to assess the clinical results of mold cup arthroplasty for traumatic hip arthritis. It made it possible for surgeons to compare their surgical outcomes in the literature. And it is the most widely used physician-assessed measurement of hip function after total hip arthroplasty.

- Interrater reliability of a modified Ashworth scale of muscle spasticity [24] with *C*_2016_ of 197 and *TC*_2016_ of 2169.

The modified Ashworth scale is the most common clinical scale used to measure the increase of muscle tone and to monitor the course of disease. It was the first time that the concept of “Modified Ashworth Scale” had been proposed and that “grade 1+” had been added in the definitions. Meanwhile, the authors graded the elbow flexor muscle spasticity of 30 patients with intracranial lesions and proved the reliability of “modified Ashworth scale.”

- Rationale of the knee society clinical rating system [25] with *C*_2016_ of 161 and *TC*_2016_ of 2161.

This paper presented a newly developed rating system for the knee. The knee society clinical rating system has been widely validated. The unified usage of it allows clinicians across the world to objectively compare their operational outcomes.

**Fig. 3:**
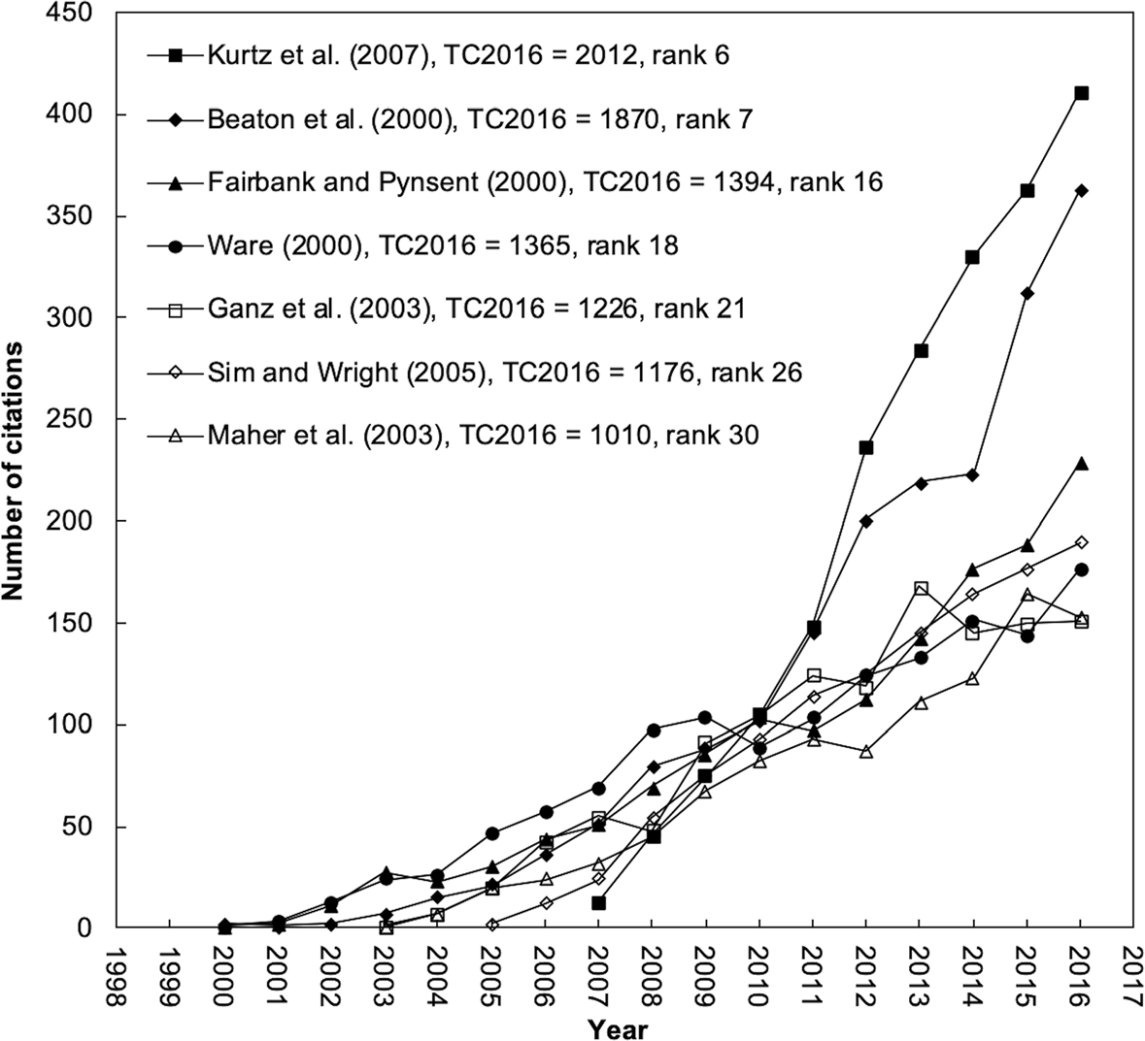
Seven classic articles with sharp increasing citation trend

Figure 3 shows trends of seven classic articles with sharp increasing in citations. These articles might be high impact in WOS category of orthopedics. In addition, classic author J.E. Ware also published the three classic articles about MOS 36-Item short-form [53–55].

### Classic sleeping beauties in web of science category of orthopedics

A “sleeping beauty” is a term that describes a research article that remains relatively uncited for a time and then suddenly bursts out. Van Raan [12, 56] defined the three characteristics of “sleeping beauties” to be depth of sleep, length of sleep, and awakening intensity.

1. The depth of sleep, where an article receives at most one citation on average per year (deep sleep), or between one to two citations per year during a specific period (less deep sleep)
2. The length of sleep—the duration of the above period
3. The intensity of the wakeup period: the number of citations per year for 4 years following the sleeping period

**Fig. 4:**
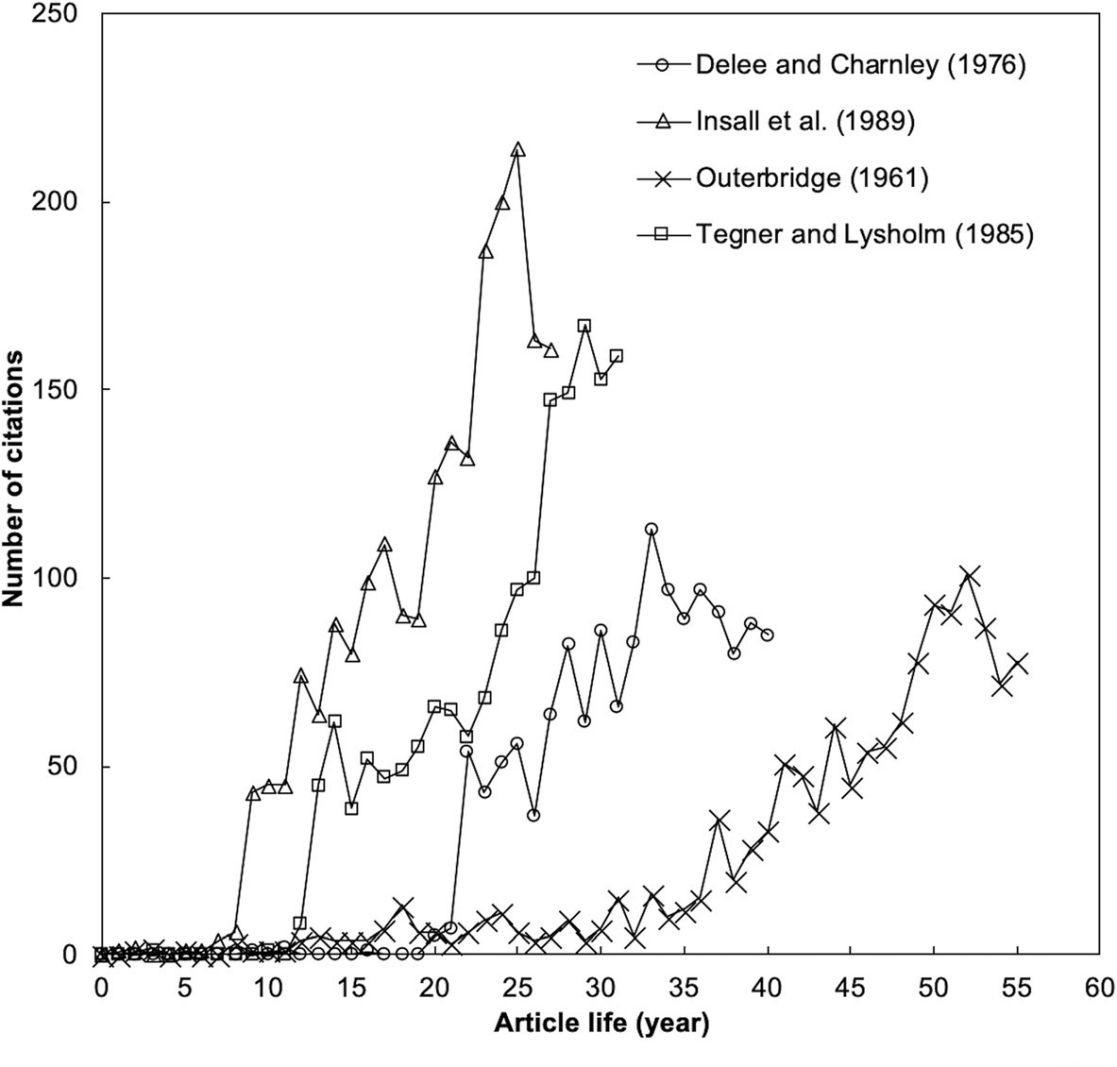
Four high impact sleeping beauty lives

Furthermore, long sleep and high impact sleeping beauties were also discussed [12]. Table 5 lists six high impact sleeping beauties in Web of Science category of orthopedics [12]. Figure 4 shows typical citation curves for four of them. The life of the article by Delee and Charnley [36] shown in Fig. 3 had the longest sleeping period with the deep sleep and the less deep sleep of 22 years respectively. The article by Outerbridge [21] was the earliest sleeping beauty while the article by Insall et al. [25] was the latest one in Web of Science category of orthopedics. Articles by Insall et al. [25] and Tegner and Lysholm [34] had higher impact in recent year. Furthermore, the article by Tegner and Lysholm kept in a plateau for 7 years after its sleep for 13 years and then “wake up” again to reach a high position in short period.

**Table 5:** Six high impact sleeping beauties in Web of Science category of orthopedics

*C*_2016_	*TC*_2016_	*L*_D_	*L*_LD_	*L*_H_	References
78	1331	17	12	36	Outerbridge (1961) [21]
85	1440	22	22	12	Delee and Charnley (1976) [36]
110	1771	19	18	11	Gruen et al. (1979) [32]
159	1674	13	13	14	Tegner and Lysholm (1985) [34]
107	2058	11	11	10	Constant and Murley (1987) [27]
161	2161	9	7	9	Insall et al. (1989) [25]

*TC*_2016_ the total citations since publication to the end of the last year (2016), *C*_2016_ the total citations in recent year (the last year 2016) only, *L*_D_ length of the deep sleep (year), *L*_LD_ length of the less deep sleep (year), *L*_H_ years to reach 100 annual citations after the less deep sleep (year)

## Conclusion

The bibliometric analysis provides a comprehensive overview of the most influential publications in the field of orthopedics. Based on our analysis, the decade with the most articles was the 1980s. All included articles belong to the document type of article and were written in English. The citation history of classic articles might serve as a guide to the understanding of the discipline.

## Data Availability

The data was all shown in the manuscript.

## Abbreviations

JCR: Journal Citation Reports
SCI-EXPANDED: Science Citation Index Expanded
UK: United Kingdom
WOS: Web of Science
